# Measurement properties of Arabic-validated sleep quality instruments: a COSMIN-based systematic review from the BRIDGE Project

**DOI:** 10.3389/fpsyg.2026.1872962

**Published:** 2026-08-18

**Authors:** Ibtissam El Harch, Oumar Abakar Mahamed, Hatem Elbanna, Soumaya Benmaamar, Hajar Mahfoudi, Nassiba Bahra, Fatima Zahrae Bartal, Moncef Maiouak, Nada Otmani, Samira El Fakir, Klaus Bös, Laura Wolbring, Mohamed Aly, Oussama Abdelkarim, Karima El Rhazi

**Affiliations:** 1 Laboratory of Epidemiology, Clinical Research and Community Health, Faculty of Medicine, Pharmacy and Dentistry, Sidi Mohamed Ben Abdellah University, Fez, Morocco; 2 Laboratory of Epidemiology and Research in Health Sciences (ERESS), Faculty of Medicine, Pharmacy and Dentistry, Sidi Mohamed Ben Abdellah University, Fez, Morocco; 3 Department of Epidemiology, Hassan II University Hospital Center, Fez, Morocco; 4 Department of Sports Psychology, Faculty of Sport Science, Mansoura University, Mansoura, Egypt; 5 Neurocognition and Action – Biomechanics Research Group, Faculty of Psychology and Sports Science, Bielefeld University, Bielefeld, Germany; 6 Institute for Sports and Sports Science, Karlsruhe Institute of Technology, Karlsruhe, Germany; 7 Faculty of Liberal Arts and Sciences, Chukyo University, Nagoya, Japan; 8 Faculty of Sport Sciences, Assiut University, Assiut, Egypt; 9 Department of Sports Management, School of Business, ESLSCA University, Giza, Egypt

**Keywords:** Arabic language, Arabs, COSMIN, cross-cultural adaptation, measurement properties, psychometric validation, psychometrics, sleep quality

## Abstract

**Background:**

Sleep quality is a multidimensional and inherently subjective construct that plays a fundamental role in physical and mental health. Numerous self-report instruments have been developed to assess subjective sleep quality; however, their cross-cultural validity must be established before use in different linguistic contexts.

**Objective:**

The objective was to systematically identify the Arabic-validated sleep quality instruments and to critically evaluate their methodological quality and psychometric properties in accordance with COSMIN standards.

**Methods:**

This systematic review was conducted following the PRISMA-COSMIN guidelines. PubMed/MEDLINE, Scopus, and Web of Science were searched from inception to December 2025. Studies reporting the translation, cross-cultural adaptation, or validation of sleep quality instruments in Arabic were included. Methodological quality was assessed using the COSMIN Risk of Bias checklist. Measurement properties were evaluated according to COSMIN criteria for good measurement properties.

**Results:**

Fifteen validation studies conducted across seven Arab countries and one general Arabic-speaking cohort were included. The Pittsburgh Sleep Quality Index was the most extensively validated instrument (*n* = 9 studies), demonstrating generally adequate structural validity but heterogeneous internal consistency results across populations. The Sleep Quality Scale (SQS) showed acceptable psychometric performance following structural adaptation. The Single-item SQS and the Jenkins Sleep Scale provided pragmatic, brief alternatives with acceptable construct validity, though limited structural evaluation. In intensive care settings, the Richards-Campbell Sleep Questionnaire and the Freedman Sleep Questionnaire Questionnaire demonstrated satisfactory internal consistency. Across instruments, structural validity and internal consistency were frequently evaluated, whereas measurement error, responsiveness, and cross-cultural measurement invariance were seldom examined.

**Conclusion:**

Multiple sleep quality instruments are available for use in Arabic-speaking populations. However, none demonstrated comprehensive evaluation across all COSMIN-recommended measurement properties. Instrument selection should be guided by both psychometric evidence and conceptual alignment with the intended context of use. Future validation studies should prioritize responsiveness, measurement error estimation, and formal cross-cultural invariance testing to strengthen the evidence base for sleep quality assessment in Arabic contexts.

**Systematic review registration:**

https://www.crd.york.ac.uk/PROSPERO/view/CRD420261328016, Identifier CRD420261328016.

## Background

Sleep is a complex biological process that extends far beyond the mere cessation of consciousness. It represents a dynamic physiological state during which the brain, although disengaged from voluntary activity, maintains regulated responsiveness to internal and external stimuli (Brinkman et al., 2025). This cycle occupies nearly one-third of human life (Browman and Winslow, 1989) and is essential for maintaining physical, psychological, and social well-being (Itani et al., 2017; Freeman et al., 2017; Hirshkowitz et al., 2015).

Sleep is now considered a multidimensional construct that is not limited to its duration, but also includes regularity, timing, continuity, efficiency, and subjective sleep quality (Katulka et al., 2020). From this perspective, sleep quality should not be regarded simply as an outcome associated with sleep, but as a constitutive dimension of sleep health.

According to the Encyclopedia of Behavioral Medicine, sleep quality is defined as “one’s satisfaction with the sleep experience, integrating aspects of sleep initiation, sleep maintenance, sleep duration, and refreshment upon awakening” (Kline, 2013). While this definition incorporates measurable parameters such as sleep latency and duration, sleep quality is fundamentally a subjective construct reflecting an individual’s overall evaluation of their sleep experience. It therefore encompasses both objective dimensions of sleep continuity and the perceived restorative value of sleep (Nelson et al., 2022).

Multiple physiological, psychological, and environmental factors influence sleep quality, including age, circadian rhythm, body mass index, stress, anxiety, depression, ambient temperature, and exposure to electronic devices. Social and occupational demands may further shape sleep perception and patterns (Nelson et al., 2022).

Good sleep quality is associated with restorative functioning, optimal cognitive performance, and emotional stability (Nelson et al., 2022). Conversely, poor sleep quality has been linked to fatigue, irritability, daytime dysfunction, impaired reaction time, increased stimulant consumption, and various adverse health outcomes, including cardiovascular disease, mental health disorders, and reduced quality of life (Sarode et al., 2023; Scott et al., 2021; Sneha et al., 2025).

Sleep quality may be assessed using objective physiological tools such as polysomnography (PSG) and actigraphy, or through subjective self-report instruments (Chen et al., 2023). Although objective methods provide precise data on sleep architecture and efficiency (Walia and Mehra, 2019), they do not fully capture the perceived experience of sleep, which lies at the core of the sleep quality construct. Self-report scales therefore play a central role in both clinical practice and research due to their feasibility, accessibility, and ability to measure subjective sleep evaluation (Chen et al., 2023; Fabbri et al., 2021). In large-scale epidemiological studies and resource-limited settings, their use becomes particularly indispensable.

Several self-report instruments have been developed to assess subjective sleep quality in both clinical and community settings. These tools differ in their conceptual foundations and structural composition. Some adopt multidimensional frameworks that integrate aspects such as sleep duration, sleep efficiency, and daytime functioning into a global score, whereas others provide a more concise assessment based on key nocturnal experiences or overall subjective evaluation (Fabbri et al., 2021). Despite these variations, all aim to capture the individual’s perception of sleep quality. The diversity of conceptual approaches underscores the importance of carefully examining the psychometric robustness of these instruments, particularly when they are adapted for use in different linguistic and cultural contexts.

The Arab-speaking countries represent a heterogeneous sociocultural and linguistic landscape, and the direct application of instruments developed in other contexts may compromise measurement validity. Sleep quality scales must therefore undergo rigorous translation and cross-cultural adaptation before being used in new settings. This process involves the evaluation of key psychometric properties to ensure that the instrument accurately measures the intended construct within the target population (DeVellis and Thorpe, 2022; Sperber et al., 1994).

Scale validation requires strict methodological standards, including forward and backward translation, expert review, cognitive testing, and formal assessment of measurement properties (Gana et al., 2021; Caron, n.d.). According to international guidelines such as the Consensus-based Standards for the Selection of Health Measurement Instruments (COSMIN), cross-cultural validation should include the evaluation of reliability, structural validity, construct validity, measurement error, and other relevant properties to ensure appropriate use across different populations (Mokkink et al., 2016).

Although several sleep-related instruments have been translated or adapted for Arabic-speaking populations, the available versions differ in their processes of cross-cultural adaptation and in the extent to which their psychometric properties have been evaluated. A systematic review is therefore needed to identify the available Arabic-language instruments, critically appraise the quality of the validation evidence, and inform researchers and clinicians about the most appropriate tools for use in Arab-speaking populations.

To the best of our knowledge, no systematic review has comprehensively synthesized the Arabic-validated sleep quality instruments according to contemporary methodological standards. Therefore, the objective of this systematic review was to identify these instruments and to critically evaluate their methodological quality and psychometric properties.

## Materials and methods

### Protocol and registration

This systematic review was conducted in accordance with the 2024 PRISMA-COSMIN guidelines (Preferred Reporting Items for Systematic Reviews and Meta-Analyses for Outcome Measurement Instruments) (Elsman et al., 2024). The review protocol was registered in the International Prospective Register of Systematic Reviews (PROSPERO; registration number: CRD420261328016).

### Search strategy

A comprehensive literature search was conducted across three major electronic databases: PubMed/MEDLINE, Scopus, and Web of Science, chosen for their broad and complementary coverage of the biomedical, psychological, and multidisciplinary literature. No restrictions on publication date were applied, ensuring that all studies published on the topic up to December 2025 were considered.

In addition to the electronic database searches, a supplementary search was performed by manually screening the reference lists of all included studies and by conducting targeted searches in Google Scholar to identify potentially relevant articles not captured by the database search.

Grey literature was not systematically searched.

The search strategy combined MeSH terms and free-text keywords related to three main concepts: (1) Sleep quality, (2) Sleep quality instruments and their psychometric properties: validation, reliability, translation, and cross-cultural adaptation, (3) Geographical context, including Arabic-speaking populations and Arab countries: Algeria, Bahrain, Comoros, Djibouti, Egypt, Iraq, Jordan, Kuwait, Lebanon, Libya, Morocco, Oman, Palestine, Qatar, Saudi Arabia, Somalia, Sudan, Syria, Tunisia, the United Arab Emirates, and Yemen (Adrian, 2019). The complete strings including all MeSH terms and free-text keywords are provided in Table 1.

**Table 1:** Searched databases and search string.

**Databases**	**Search string**
PubMed-MEDLINE	((“Sleep Quality”[Mesh] OR “Sleep Wake Disorders”[Mesh] OR “sleep quality”[tw] OR “sleep assessment”[tw] OR “sleep questionnaire”[tw] OR “sleep scale”[tw]) AND (“Arabic”[tw] OR “Arabic language”[Mesh] OR “Arabs”[Mesh] OR “Algeria”[tw] OR “Bahrain”[tw] OR “Comoros”[tw] OR “Djibouti”[tw] OR “Egypt”[tw] OR “Iraq”[tw] OR “Jordan”[tw] OR “Kuwait”[tw] OR “Lebanon”[tw] OR “Libya”[tw] OR “Morocco”[tw] OR “Oman”[tw] OR “Palestine”[tw] OR “Qatar”[tw] OR “Saudi Arabia”[tw] OR “Somalia”[tw] OR “Sudan”[tw] OR “Syria”[tw] OR “Tunisia”[tw] OR “United Arab Emirates”[tw] OR “Yemen”[tw] OR “Maghreb”[tw]) AND (“Validation”[tw] OR “Reliability”[tw] OR “Reproducibility of Results”[Mesh] OR “Psychometrics”[Mesh] OR “psychometric”[tw] OR “translation”[tw] OR “cross-cultural”[tw] OR “adaptation”[tw]))
Scopus and Web Of Science	TITLE-ABS-KEY ((“sleep quality” OR “sleep questionnaire” OR “sleep scale” OR “sleep assessment”) AND (“Arabic”) AND (“valid*” OR “reliab*” OR “psychometric” OR “translat*”))

### Eligibility criteria

Studies were selected based on the following inclusion criteria:

- *Study design:* Studies reporting the translation, cross-cultural adaptation, or validation of a specific sleep quality questionnaire in the Arabic language, including the evaluation of its psychometric properties.
- *Population:* Studies involving Arabic-speaking populations (general population or specific subgroups, clinical or community-based), regardless of country of residence.
- *Language and publication:* Original articles published in English, French, or Arabic with full text available.

*Exclusion criteria included:*

- Studies assessing sleep-related constructs other than sleep quality (e.g., sleep apnea, insomnia severity…).
- Studies using a sleep quality questionnaire solely as an outcome measure without reporting validation or psychometric evaluation of the Arabic version.
- Review articles, editorials, letters to the editor, and conference abstracts lacking sufficient methodological data.

### Study selection

All identified records were exported to Excel, and duplicates were removed. The selection process was conducted in three stages (title, abstract, and full text) by the same two independent reviewers (I.ELH. and MO. ABA). Any disagreement arising during the process was resolved through consensus or by the arbitration of a third reviewer (H.ELB).

### Data extraction

Data extraction was performed independently by the same two reviewers (I.ELH. and MO. ABA) using a standardized form aligned with PRISMA-COSMIN recommendations (Elsman et al., 2024). Extracted information included:

- *General information:* Authors, year, country, and original language of the instrument.
- *Sample characteristics:* Target population, sample size, gender distribution, and mean age.
- *Instrument characteristics:* Name of the questionnaire, number of items and dimensions, and language of validation (Modern Standard Arabic or dialect).
- *Psychometric properties:* Content validity, structural validity (factor analysis), internal consistency (Cronbach’s alpha), test–retest reliability, construct validity, criterion validity, measurement error and cross-cultural validity.

When multiple studies evaluated the same instrument, each validation study was assessed independently for methodological quality and measurement properties. Findings were then qualitatively synthesized per instrument and per measurement property in accordance with COSMIN recommendations.

### Methodological quality assessment (risk of bias)

The methodological quality of the included studies was assessed using the COSMIN Risk of Bias checklist in accordance with the updated COSMIN guidelines (2018/2020) (Terwee et al., 2012). This standardized tool is specifically designed to evaluate the methodological quality of studies on measurement properties of patient-reported outcome measures and other health-related instruments.

### Assessment procedure

Each included study was independently evaluated across the 10 measurement property domains defined by the COSMIN Risk of Bias checklist (Terwee et al., 2012):

1. *Instrument development:* Evaluates the quality of the instrument’s initial design, specifically the item generation process and qualitative pilot studies conducted to ensure items reflect the construct of interest.
2. *Content validity:* Assesses whether the instrument’s content (items, instructions) is relevant, comprehensive, and comprehensible for both the target population and professionals.
3. *Structural validity:* Verifies whether the internal structure of the scale (dimensions/factors) is confirmed by appropriate methods (Factor analysis or Rasch models) and aligns with the underlying theory.
4. *Internal consistency:* Measures the degree of interrelatedness among items within the same dimension, typically assessed using Cronbach’s alpha.
5. *Cross-cultural validity/measurement invariance:* Evaluates the quality of the translation and cultural adaptation process, as well as the measurement invariance compared to the original version.
6. *Reliability:* Estimates the stability of scores over time (test–retest reliability) in patients whose clinical status has not changed.
7. Measurement error: Assesses the systematic and random error of a patient’s score that is not attributed to true changes (e.g., Standard Error of Measurement—SEM).
8. *Criterion validity:* Determines the extent to which the instrument’s scores adequately reflect a recognized “Gold Standard.
9. *Construct validity (hypothesis testing):* Tests whether the instrument behaves as expected in relation to other instruments (convergent/divergent validity) or between known groups (discriminative validity).
10. *Responsiveness:* Evaluates the instrument’s ability to detect clinically important changes over time.

Each domain (referred to as a “box” in the COSMIN framework) contains multiple methodological standards related to study design and statistical analysis, which were assessed separately for each measurement property reported.

### Rating and scoring system

In accordance with COSMIN recommendations, the “worst score counts” principle was applied. Each methodological standard within a measurement property domain was rated using a four-point scale: Very good, Adequate, Doubtful, or Inadequate, and the lowest rating determined the overall methodological quality for that measurement property (Terwee et al., 2012).

When a measurement property was not evaluated within a study, no COSMIN rating was assigned for that property. When a measurement property was not applicable to the study design or to the characteristics of the instrument, it was classified as not applicable (NA), in line with COSMIN guidance (Terwee et al., 2012).

Measurement properties that were not evaluated or deemed not applicable were not rated and were therefore not included in the methodological quality assessment for that specific study (Terwee et al., 2012).

### Evaluation of measurement properties (COSMIN criteria)

The results of the measurement properties were evaluated using the COSMIN criteria for good measurement properties (Mokkink, n.d.). For each measurement property, reported psychometric results were rated as sufficient (+), insufficient (−), or indeterminate (?), according to predefined COSMIN thresholds. This evaluation was conducted independently of the methodological quality assessment (Mokkink, n.d.).

## Results

### Search results

The electronic search strategy identified a total of 276 records: 100 from Web of Science, 74 from Scopus, and 102 from PubMed/MEDLINE. Following the removal of 44 duplicates, 232 unique records were screened.

Based on title and abstract screening, 213 records were excluded (185 based on title and 28 based on abstract), leaving 19 articles for full-text assessment. Of these, six were excluded for the following reasons: the scale did not measure sleep quality (*n* = 2) or the full text was not available (*n* = 4).

In addition, two further studies were identified through manual searching of the reference lists of included articles and through Google Scholar.

**Figure 1:**
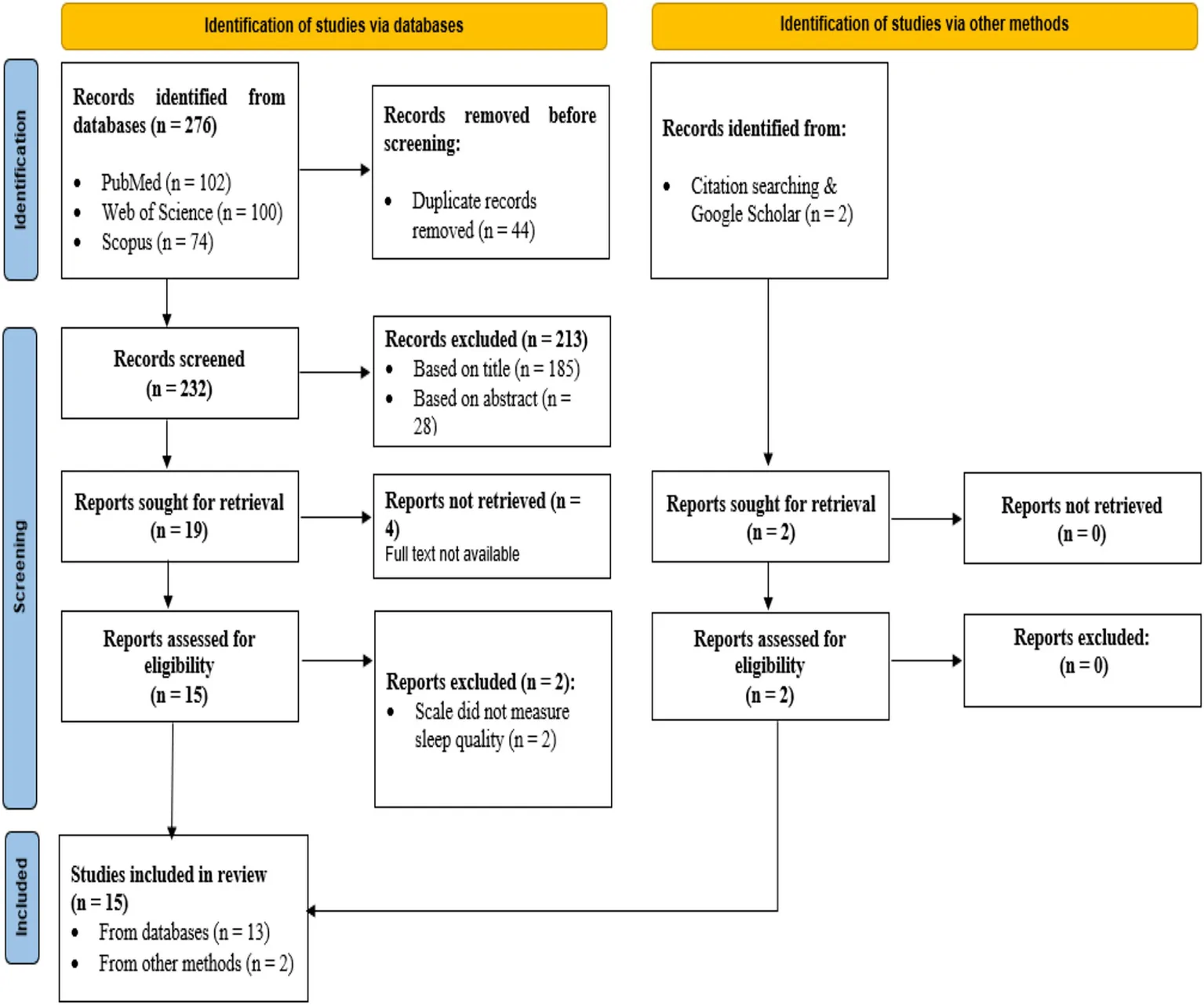
PRISMA flow diagram of the study selection process.

Ultimately, 15 studies met the inclusion criteria and were included in this systematic review. The study selection process is illustrated in the PRISMA flow diagram (Figure 1).

### Characteristics of included studies

**Figure 2:**
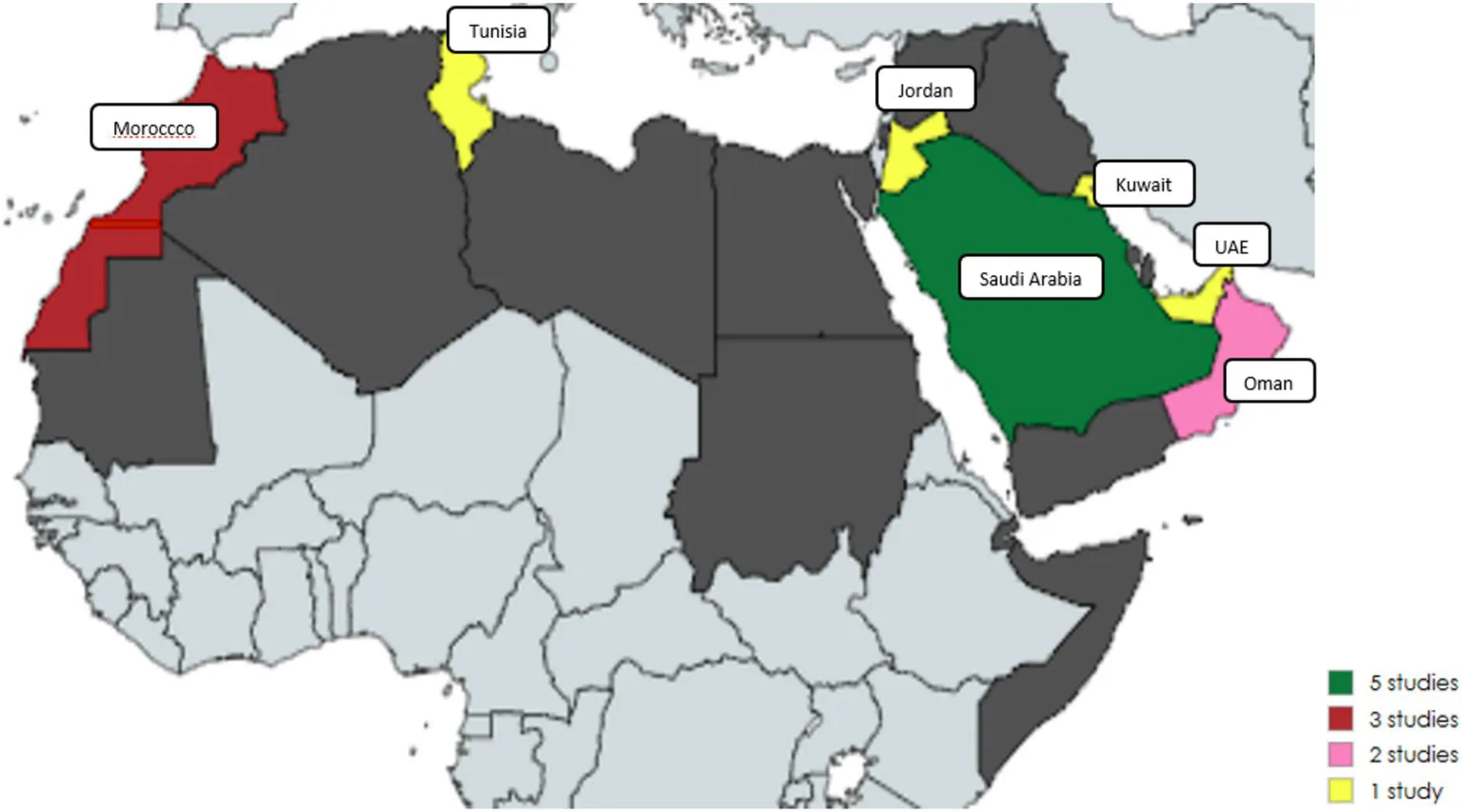
Geographical distribution of the included studies.

The included articles were published between 2012 and 2025, covering seven specific countries and one general Arabic population: Saudi Arabia (*n* = 5), Morocco (*n* = 3), Oman (*n* = 2), Jordan (*n* = 1), Kuwait (*n* = 1), Tunisia (*n* = 1), and the United Arab Emirates (UAE) (*n* = 1), alongside one study targeting the general Arabic-speaking population (Figure 2).

Across the 15 included studies, six distinct sleep quality instruments were culturally adapted and validated. The Pittsburgh Sleep Quality Index (PSQI) was the most frequently evaluated scale (*n* = 9) (Malik et al., 2018; Alqarni et al., 2025; Aldhahi et al., 2026; Suleiman et al., 2012; Al Maqbali et al., 2020; Albatineh et al., 2022; Oneib et al., 2023; Ben Letaifa et al., 2024; Arumugam et al., 2024). The remaining studies evaluated the Sleep Quality Scale (SQS) (*n* = 1) (Alghamdi, 2025), a Single-item SQS (*n* = 1) (Badahdah et al., 2024), the Jenkins Sleep Scale (JSS) (*n* = 1) (AlMashouk et al., 2024), the Richards-Campbell Sleep Questionnaire (RCSQ) (*n* = 2) (Al-Sulami et al., 2019; Lkoul et al., 2025a), and the Freedman Sleep Questionnaire (FSQ) (*n* = 1) (Lkoul et al., 2025b) (Table 1).

The total pooled sample size comprised 4,408 participants, with individual study samples ranging from 50 (in the UAE study) to 560 (in the Tunisian study) (Table 1).

(1) *PSQI*

The PSQI was the most frequently validated scale. This 19-item self-report instrument was evaluated in nine studies assessing the Arabic version of the scale in various countries: Saudi Arabia (*n* = 3) (Malik et al., 2018; Alqarni et al., 2025; Aldhahi et al., 2026), Jordan (*n* = 1) (Suleiman et al., 2012), Oman (*n* = 1) (Al Maqbali et al., 2020), Kuwait (*n* = 1) (Albatineh et al., 2022), Morocco (*n* = 1) (Oneib et al., 2023), Tunisia (*n* = 1) (Ben Letaifa et al., 2024), and the United Arab Emirates (*n* = 1) (Arumugam et al., 2024).

The populations were heterogeneous, encompassing healthy general populations (Aldhahi et al., 2026), adolescents and students (Alqarni et al., 2025; Oneib et al., 2023; Ben Letaifa et al., 2024; Arumugam et al., 2024), clinical groups including patients with coronary artery disease (Suleiman et al., 2012), cancer (Al Maqbali et al., 2020), and those undergoing hemodialysis (Albatineh et al., 2022), as well as healthcare professionals (Malik et al., 2018). Accordingly, reported age measures varied considerably across studies, from a mean age of 13 years in Tunisian adolescents (Ben Letaifa et al., 2024) to a median age of 54 years in Kuwaiti hemodialysis patients (Albatineh et al., 2022). Gender distribution also varied, with most studies reporting a predominance of female participants (Table 2).

**Table 2:** General characteristics of the included studies and the validated sleep quality instruments.

Authors and year of validation	Original instrument name	Original language	Validation country	Arabic linguistic variety	Construct measured	Number of instrument items and subscales	Target population	Sample size	Mean age (years)	Percentage male (%)
Suleiman et al. (2012)	PSQI	English	Jordan	Modern Standard Arabic	Sleep quality	-Items: 19 -Subscales: 7	Coronary artery disease patients	130	46.7 ± 14.73	50.7
Malik et al. (2018)	PSQI	English	Saudi Arabia	Modern Standard Arabic	Sleep quality	-Items: 19 -Subscales: 7	Physicians working in Jeddah health facilities	330	The most frequently observed category of Age: 23–30 years (32.73%)	55.76
Alqarni et al. (2025)	PSQI	English	Saudi Arabia	Modern Standard Arabic	Sleep quality	-Items: 19 -Subscales: 7	undergraduate medical students (from the first to the fifth year) and medical interns	202	Not reported	43.1
Aldhahi et al. (2026)	PSQI	English	Saudi Arabia	Modern Standard Arabic	Sleep quality	-Items: 19 -Subscales: 7	healthy adult participants	314	29.14 ± 12.0	17.8
Al Maqbali et al. (2020)	PSQI	English	Oman	Modern Standard Arabic	Sleep quality	-Items: 19 -Subscales: 7	Cancer patients	369	The most frequently observed category of Age: 41–50 years (25.7%)	33.6
Albatineh et al. (2022)	PSQI	English	Kuwait	Modern Standard Arabic	Sleep quality	-Items: 19 -Subscales: 7	Hemodialysis patients	461	median (Interquartile Range): 54 (22) years	62.3
Oneib et al. (2023)	PSQI	English	Morocco	Moroccan Dialect	Sleep quality	-Items: 19 -Subscales: 7	Medical students and medical and nursing staff	120	29.23 ± 6.69	40.8
Ben Letaifa et al. (2024)	PSQI	English	Tunisia	Tunisian Arabic	Sleep quality	-Items: 19 -Subscales: 7	Middle school students	560	13	47.0
Arumugam et al. (2024)	PSQI	English	UAE	Modern Standard Arabic	Sleep quality	-Items: 19 -Subscales: 7	adolescents and young adults	50	20.82 ± 2.7	32.0
Alghamdi (2025)	SQS	English	Saudi Arabia	Modern Standard Arabic	Sleep quality	-Items: 28 -Subscales: 6	Adult Saudi population	402	33.76 ± 8.37	66.1
Badahdah et al. (2024)	Single-item SQS	English	Oman	Modern Standard Arabic	Sleep quality	-Items: 1	Healthcare providers in Oman	509	37.67 ± 7,68	19.7
AlMashouk et al. (2024)	JSS	English	Arabic population	Modern Standard Arabic	Sleep quality	Items: 4 Subscales: 0	Arabic-speaking Adult	420	22.66 ± 6.85	17%
Al-Sulami et al. (2019)	RCSQ	English	Saudi Arabia	Modern Standard Arabic	Sleep quality of critically ill patients	Items: 5 Subscales: 0	critically ill adult patients at the University Hospital in Jeddah city	57	54	61.0
Lkoul et al. (2025a)	RCSQ	English	Morocco	Moroccan Dialect	Sleep quality of critically ill patients	Items: 5 Subscales: 0	Moroccan critically ill patients from three ICUs in three hospitals in the Souss-Massa region in southern Morocco	224	49 ± 18.3	47.76
Lkoul et al. (2025b)	FSQ	English	Morocco	Moroccan Dialect	Subjective sleep quality of critically ill patients	Items: 27 Subscales: 4	Moroccan patients admitted to the ICU in three hospitals in the Souss-Massa region	260	49.6 ± 18.42	46.6

The methodological quality of the PSQI validation studies varied considerably depending on the specific measurement property assessed.

Content validity was evaluated in four studies (Al Maqbali et al., 2020; Albatineh et al., 2022; Oneib et al., 2023; Ben Letaifa et al., 2024). The methodological quality of these evaluations was generally limited and rated as “Doubtful” in Omani cancer patients (Al Maqbali et al., 2020), Kuwaiti hemodialysis patients (Albatineh et al., 2022), and Moroccan healthcare populations (Oneib et al., 2023), and “Inadequate” in Tunisian students (Ben Letaifa et al., 2024). Despite these methodological limitations, the translation and cultural adaptation procedures resulted in items considered clear and relevant for the target populations. Consequently, the overall result for content validity was rated as sufficient (+).

Structural validity was assessed in six studies (Malik et al., 2018; Aldhahi et al., 2026; Al Maqbali et al., 2020; Albatineh et al., 2022; Oneib et al., 2023; Ben Letaifa et al., 2024). The methodological quality was rated as “Very Good” in five of these studies (Malik et al., 2018; Aldhahi et al., 2026; Al Maqbali et al., 2020; Albatineh et al., 2022; Oneib et al., 2023), and “Adequate” in the Tunisian study (Ben Letaifa et al., 2024) that relied exclusively on exploratory factor analysis (EFA) without confirmatory analysis (CFA). Factor analytic approaches, including CFA and Rasch modeling, supported either a unidimensional structure or a two-factor structure, often demonstrating improved model fit compared with the original component model. This property was rated as sufficient (+) in five studies (Malik et al., 2018; Aldhahi et al., 2026; Al Maqbali et al., 2020; Albatineh et al., 2022; Ben Letaifa et al., 2024). However, one study (Oneib et al., 2023) was rated as insufficient (−) although certain fit indices were satisfactory (RMSEA = 0.06, SRMR = 0.05), key values (CFI = 0.94, TLI = 0.93) were very close to, but did not reach, the strict >0.95 threshold set by COSMIN.

Internal consistency was evaluated in all nine validation studies, and the methodological quality was rated as “Very Good.” Reported alpha coefficients ranged from 0.56 (Alqarni et al., 2025) to 0.77 (Al Maqbali et al., 2020), indicating heterogeneous reliability across populations. Based on the COSMIN threshold of *α* ≥ 0.70, three studies demonstrated sufficient internal consistency (+) [Jordanian patients with coronary artery disease (Suleiman et al., 2012), Saudi physicians (Malik et al., 2018), and Omani cancer patients (Al Maqbali et al., 2020).] while six studies (Alqarni et al., 2025; Aldhahi et al., 2026; Albatineh et al., 2022; Oneib et al., 2023; Ben Letaifa et al., 2024; Arumugam et al., 2024) reported values below the recommended threshold and were rated as insufficient (−).

Test–retest reliability was evaluated in three studies (Alqarni et al., 2025; Oneib et al., 2023; Arumugam et al., 2024), all of which demonstrated “Very Good” methodological quality. Intraclass correlation coefficients (ICC) ranged from 0.61 to 0.77. Two studies (Alqarni et al., 2025; Arumugam et al., 2024) reported ICC values above the COSMIN threshold (ICC ≥ 0.70) and were therefore rated as sufficient (+), while one study (Oneib et al., 2023) reported an ICC of 0.61 and was rated as insufficient (−).

Construct validity was evaluated in four studies with “Very Good” methodological quality (Suleiman et al., 2012; Al Maqbali et al., 2020; Albatineh et al., 2022; Oneib et al., 2023). Three studies (Suleiman et al., 2012; Al Maqbali et al., 2020; Oneib et al., 2023) demonstrated expected correlations with related instruments or successfully differentiated relevant clinical subgroups, resulting in a sufficient (+) rating. One study did not formulate predefined hypotheses to interpret correlations, resulting in an indeterminate (?) rating (Albatineh et al., 2022).

Cross-cultural validity was evaluated in one study using Rasch analysis (Aldhahi et al., 2026). The methodological quality was rated as “Very Good.” However, the analysis revealed significant differential item functioning for the sleep duration component across gender and age groups, leading to an insufficient (−) rating for this property.

Measurement error was evaluated in only one study involving Emirati adolescents and young adults (Arumugam et al., 2024). The methodological quality was rated as “Very Good.” However, because no MIC was defined, measurement error could not be interpreted and was rated as indeterminate (Tables 3–5).

(2) *The SQS*

**Table 3:** Evaluation of the methodological quality of validation studies based on the COSMIN checklist bias.

First Author, Year (Country)	PROM development	Content validity	Structural validity	Internal consistency	Cross-cultural validity	Reliability	Measurement error	Criterion validity	construct validity	Responsiveness
Suleiman et al. (2012)	NA	NE	NE	Very good	NE	NE	NE	NE	Very good	NE
Malik et al. (2018)	NA	NE	Very good	Very good	NE	NE	NE	NE	NE	NE
Alqarni et al. (2025)	NA	NE	NE	Very good	NE	Very good	NE	NE	NE	NE
Aldhahi et al. (2026)	NA	NE	Very good	Very good	Very good	NE	NE	NE	NE	NE
Al Maqbali et al. (2020)	NA	Doubtful	Very good	Very good	NE	NE	NE	NE	Very good	NE
Albatineh et al. (2022)	NA	Doubtful	Very good	Very good	NE	NE	NE	NE	Very good	NE
Oneib et al. (2023)	NA	Doubtful	Very good	Very good	NE	Very good	NE	NE	Very good	NE
Ben Letaifa et al. (2024)	NA	Inadequate	Adequate	Very good	NE	NE	NE	NE	NE	NE
Arumugam et al. (2024)	NA	NE	NE	Very good	NE	Very good	Very good	NE	NE	NE
Alghamdi (2025)	NA	Very good	Very good	Very good	NE	Very good	NE	NE	NE	NE
Badahdah et al. (2024)	NA	NE	NE	NE	NE	NE	NE	NE	Very good	NE
AlMashouk et al. (2024)	NA	Very good	Very good	Very good	NE	Very good	NE	NE	Very good	NE
Al-Sulami et al. (2019)	NA	Very good	Inadequate	Very good	NE	NE	NE	NE	NE	NE
Lkoul et al. (2025a)	NA	Very good	Adequate	Very good	NE	Very good	NE	NE	Very good	NE
Lkoul et al. (2025b)	NA	Very good	Adequate	Very good	NE	Very good	NE	NE	NE	NE

NE, not evaluated; NA, not applicable.

**Table 4:** Psychometric characteristics of the validated measurement scales.

**First Author, Year (Country)**	**Instrument**	**Acceptability**	**Floor and ceiling effect**	**Content validity**	**Internal Consistency**	**Reliability (Test-Retest / ICC)**	**criterion validity**	**Structural Validity (Factor Analysis)**	**Measurement error**	**Cross‐cultural validity** **(measurement invariance)**	**Construct / Concurrent Validity**
Suleiman et al. (2012)	AJ-PSQI	Not reported	Not reported	Not reported	- Cronbach's α: 0,74 - Item-to-total correlation coefficient: 0.25 - 0.49 - Item-to-item correlation: -0.24 - 0.33	Not reported	Not reported	Not reported	Not reported	Not reported	-ISI scores: r=0.36 / p<0.001 -MOS SF-36 pain subscale: r= -0.19 / p=0.032
Malik et al. (2018)	PSQI	Not reported	Not reported	Not reported	- Cronbach's α: 0.745	Not reported	Not reported	**EFA:** Bartlett's Test of Sphericity p<0.001, Measure of Sampling Adequacy (KMO)=0.771; **CFA**: NFI: >0.95; TLI: >0.95; CFI: >0.95; RMSEA: 0.06-0.09; SRMR <0.08; Normed - χ2 <3)	Not reported	Not reported	Not reported
Alqarni et al. (2025)	A-PSQI	Not reported	Not reported	Not reported	- Cronbach's α: 0.560	ICC (2 weeks): 0.711	Not reported	Not reported	Not reported	Not reported	Not reported
Aldhahi et al. (2026)	PSQI	Not reported	Most participants scored below zero logits.	Not reported	Person Reliability: 0.48.	Not reported	Not reported	-PSQI appeared to be unidimensional -PCA conducted on the model’s residuals revealed no component with an eigenvalue greater than 2.0" -Fit to the Rasch model was satisfactory for all seven components -Components 3, 4, and 6 had disordered modal thresholds.	Not reported	Differential item functioning (DIF) was calculated: -Component 3 (Sleep duration) was affected by DIF for both gender and age. -No DIF was found for physical activity.	Not reported
Al Maqbali et al. (2020)	AO-PSQI	Not reported	No floor or ceiling for the global scale - Ceiling effects were observed in subjective sleep quality, sleep latency, sleep duration and habitual sleep efficiency. -Floor effects were apparent in the use of sleep medication and daytime dysfunction.	-Translation review by two independent bilingual translators; -Pre-test on cancer patients (n=10) to examine clarity and comprehensibility; -Layout modification incorporating all questions into a table format.	-Cronbach’s alpha: 0.77 -Component-total correlation coefficient: 0.38 - 0.62	Not reported	Not reported	CAF: -A one-factor model was tested and indicate the poor fit of the model. - The two-factor model performed better fit: χ2(dl = 13) = 35.18, P < 0.001, GFI = 0.97, AGFI = 0.94, CFI = 0.97, TLI = 0.95, RMSEA = 0.06. and χ2/dl = 2.7)	Not reported	Not reported	Differences between the mean of the global PSQI according to the gender, cancer site, cancer stage and comorbidities was conducted
Albatineh et al. (2022)	AK-PSQI	Not reported	No floor or ceiling Effects are reported	-Pre-test on hemodialysis patients (n=15) to verify item clarity; -Patient and expert feedback incorporated into the final version.	- Cronbach's α: 0,634	Not reported	Not reported	**EFA (PCA) + and oblimin non-orthogonal rotation:** 2-factor structure accounting for 51.8% of the total variance KMO: 0.68 **CFA**: NFI: 0,943; CFI: 0,967; RMSEA: 0,050; SRMR 0,039.	Not reported	Not reported	Ak-PSQI mean score was significantly higher among those “can lie-down without problems” (mean = 6.5, SD = 3.5) compared to those “must raise bedhead to breath” (mean = 8.4, SD = 3.7
Oneib et al. (2023)	AM-PSQI	Not reported	Not reported	-Forward-backward translation according to Guillemin’s guidelines; -Expert committee review; -Pre-test on individuals of varying social and educational backgrounds (n=10) to assess item clarity and ambiguity; -Minor modifications incorporated into the final Moroccan dialect version.	-Cronbach’s alpha: 0.6 -Component-total correlation coefficient: 0.302 - 0.481	ICC (10 days): 0.61 (95% CI 0.491–0.707)	Not reported	CAF: -CFI = 0.94, -TLI = 0.93, -RMSEA = 0.06. -SRMR = 0.05	Not reported	Not reported	correlation between the ISI and PSQI total scores using the Pearson’s correlation coefficient: r = 0,66, p < 0,001
Ben Letaifa et al. (2024)	AT-PSQI	Response rate: 97.9%	Not reported	-Forward-backward translation; expert panel review (n=4); -Cultural adaptation of item 8; online - Pre-test by a quantitative online survey (n=228 adolescents) leading to item 9 modification.	- Cronbach's α: 0.6 - Item-to-total spearman correlation coefficient ≥ 0.464	Not reported	Not reported	EFA (PCA): - 1-factor structure (Explaining 30.3% of variance) - KMO>0,6 - Bartlett's Test of Sphericity: p<0,0005	Not reported	Not reported	Not reported
Arumugam et al. (2024)	AE-PSQI	Not reported	No floor or ceiling Effects are reported	Not reported	- Cronbach's α: 0.65 - Item-to-total Pearson correlation coefficient: 0.31 to 0.74	ICC (7 days): 0.77 (95% CI 0.63–0.87)	Not reported	Not reported	-Standard Error of Measurement (SEM): 1.6; -Smallest Real Difference (SRD: 4.5; -Bland-Altman Plots tests difference: 1.0 to 1.49 SDs	Not reported	Not reported
Alghamdi et al. (2025)	A-SQS	Not reported	Not reported	-Brislin's Model; Expert review (n=4) and pilot test (n=30) confirmed clarity.	- Cronbach's α: 0.90 Ranging between 0.588 – 0.889	ICC (15 days / n= 83): 0.88	Not reported	**EFA:** -5-factor model supported. - Item 10 removed. **CFA:** CFI=0.934, RMSEA=0.033. TFI: 0.962	Not reported	Not reported	Not reported
Badahdah et al. (2025)	A-Single-item SQS	Not reported	Not reported	Not reported	Not reported	Not reported	Not reported	Not reported	Not reported	Not reported	The SQS correlated positively with the WHO-5 (r = .48) and negatively with the GAD-7 (r = − .47) and IUS-12 (r = − .34).
AlMashou et al. (2024)	A-JSS	- % of missing data: 0%	Not reported	Forward-backward translation; expert committee (kappa > 0.99); pilot test (n=30) confirmed item clarity.	- Cronbach's α: 0.74 - McDonald’s omega: 0.75.	-ICC (2-4 weeks / n= 147): 0.94	Not reported	CFA: the maximum likelihood method confirmed the unidimensional structure of the scale -Chi² = 7.66, -p = 0.022; -CFI = 0.99; -TLI = 0.96; -RMSEA = 0.08 [95% CI: 0.03–0.15]	Not reported	Not reported	Pearson correlations with PSQI (r = 0.80, p < 0.001) and AIS (r = 0.74, p < 0.001). Predefined hypotheses were fully met
Al-Sulami et al. (2019)	A-RCSQ	-Administration time: 2 to 3 min - % of missing data: 0%	Not reported	WHO translation guidelines; cognitive debriefing interviews ($n=30$ ICU patients) confirmed excellent item clarity and comprehensibility with no reported ambiguities.	- Cronbach's α: 0.89	Not reported	Not reported	Not reported	Not reported	Not reported	Not reported
Lkoul et al. (2025a)	AM-RCSQ	- % of missing data: 0%	Not reported	The degree of convergence of the experts’ answers during the final validation was measured by the Aiken coefficient, V = 0.82. Furthermore, a cognitive debriefing with four specialists and a pilot test on 12 ICU patients confirmed that the translated items were unambiguous and clearly understood by the target population.	-Cronbach's α: 0.960	ICC (6 hours): 0.978 (0.936–0.992)	Not reported	EFA: The principal component analysis identified a single factor accounting for 84.4% of the total variance.	Not reported	Not reported	The RCSQ total score demonstrated a strong, statistically significant inverse correlation with the PSQI total score (r = -0.635, p < 0.01). Similarly, all AM-RCSQ individual items correlated significantly with their corresponding PSQI variables (r ranging from -0.551 to -0.598, p < 0.01). Known-Groups Validity: The scale successfully discriminated between individuals with poor and good sleep quality based on clinical characteristics. Poor sleepers were significantly older (p < 0.001), reported higher pain levels (p < 0.001), and had a higher prevalence of anxiety (p= 0.017) compared to good sleepers. No significant difference was observed for gender (p= 0.315).
Lkoul et al.(2025b)	AM-FSQ	Not reported	Not reported	The degree of convergence of the experts’ answers during the final validation was measured by the Aiken coefficient, V = 0.78.	Cronbach's α: 0.816	ICC: 0.715 - 0.997	Not reported	EFA: 4-factor structure (Explaining 70.8% of variance)	Not reported	Not reported	Not reported

**Table 5:** Evaluation of measurement properties (COSMIN criteria).

First Author, Year (Country)	Measurement property								
	Content validity	Structural validity	Internal consistency	Cross-cultural validity\measurement invariance	Reliability	Measurement error	Criterion validity	Hypotheses testing for construct validity	Responsiveness
Suleiman et al. (2012)	?	?	+	?	?	?	?	+	?
Malik et al. (2018)	?	+	+	?	?	?	?	?	?
Alqarni et al. (2025)	?	?	−	?	+	?	?	?	?
Aldhahi et al. (2026)	?	+	−	−	?	?	?	?	?
Al Maqbali et al. (2020)	+	+	+	?	?	?	?	+	?
Albatineh et al. (2022)	+	+	−	?	?	?	?	?	?
Oneib et al. (2023)	+	−	−	?	−	?	?	+	?
Ben Letaifa et al. (2024)	+	+	−	?	?	?	?	?	?
Arumugam et al. (2024)	?	?	−	?	+	?	?	?	?
Alghamdi (2025)	+	+	−	?	+	?	?	?	?
Badahdah et al. (2024)	?	?	?	?	?	?	?	+	?
AlMashouk et al. (2024)	+	+	+	?	+	?	?	+	?
Al-Sulami et al. (2019)	+	?	+	?	?	?	?	?	?
Lkoul et al. (2025a)	+	+	+	?	+	?	?	+	?
Lkoul et al. (2025b)	+	+	+	?	+	?	?	?	?

Green box = Sufficient (+), Red box = Insufficient (-), Yellow box = Indeterminate (?).

One study validated the Arabic version of the SQS in Saudi Arabia (Alghamdi, 2025). The SQS is a 28-item instrument designed to assess multiple dimensions of sleep quality (Yi et al., 2006). The validation study included 402 participants from the adult Saudi population (mean age: 33.76 ± 8.37 years), of whom 66.1% were male (Table 2).

The methodological quality of content validity was rated as “Very Good,” and the resulting evidence was rated as sufficient (+).

Structural validity was assessed using factor analysis with “Very Good” methodological quality. The analyses supported a stable multidimensional structure with acceptable model fit, resulting in a sufficient (+) rating.

Internal consistency showed Cronbach’s alpha values ranging from 0.58 to 0.88 across the five subscales and was rated as insufficient (−) for some subscales.

Test–retest reliability was assessed over a 15-day interval in 38 participants with “Very Good” methodological quality. The total score demonstrated strong temporal stability (*r* = 0.88), and subscale correlations ranged from 0.84 to 0.96, resulting in a sufficient (+) rating (Tables 3–5).

(3) *The single item SQS*

One study validated the Arabic version of the Single-item SQS in Oman (Badahdah et al., 2024). This instrument consists of a single question designed to capture overall perceived sleep quality. The validation study included 509 healthcare providers, with a mean age of 37.67 ± 7.68 years. The sample was predominantly female, with males representing 19.7% of participants (Table 2).

Construct validity was the only measurement property evaluated in the included study. The methodological quality of this assessment was rated as “Very Good,” as appropriate statistical analyses were conducted in a large sample. The results demonstrated correlations with related psychological constructs in the expected directions, supporting the theoretical validity of the measure. Accordingly, construct validity was rated as sufficient (+) according to COSMIN criteria (Tables 3–5).

(4) *The JSS*

One study validated the Arabic version of the JSS among the general Arabic population (AlMashouk et al., 2024). The JSS is a brief 4-item instrument without subscales designed to assess perceived sleep quality. The validation study included 420 participants, with a mean age of 22.66 ± 6.85 years, and 17% of participants were male (Table 2).

Content validity was evaluated through forward–backward translation, expert review, and pilot testing, and the methodological quality was rated as “Very Good.” Expert agreement and pilot testing confirmed item clarity, resulting in a sufficient (+) rating.

Structural validity was assessed using CFA to test the expected unidimensional structure of the scale. The methodological quality was rated as “Very Good.” All four items demonstrated adequate factor loadings (> 0.40), supporting the predefined structure of the instrument. Structural validity was therefore rated as sufficient (+).

Internal consistency showed “Adequate” reliability (*α* = 0.74; *ω* = 0.75) and was rated as sufficient (+).

Test–retest reliability was assessed over a two- to four-week interval in a subsample of 147 participants, and the methodological quality was rated as “Very Good.” The scale demonstrated excellent temporal stability (ICC = 0.94), which exceeds the COSMIN threshold for sufficient reliability. Therefore, test–retest reliability was rated as sufficient (+).

Construct validity was evaluated by testing predefined hypotheses regarding correlations with established sleep instruments. The methodological quality of this assessment was rated as “Very Good.” Strong positive correlations were observed with both the Athens Insomnia Scale (AIS) (*r* = 0.74) and the PSQI (*r* = 0.80), supporting the expected relationships between the instruments. Consequently, construct validity was rated as sufficient (+) (Tables 3–5).

(5) *Instruments validated for Intensive Care Units (ICU)*

Three studies validated Arabic versions of sleep quality instruments specifically designed for patients admitted to ICU. Two studies validated the Arabic version of the Richards-Campbell Sleep Questionnaire (RCSQ) in Saudi Arabia (Al-Sulami et al., 2019) and Morocco (Lkoul et al., 2025a), while one study validated the FSQ in Morocco (Lkoul et al., 2025b).

The RCSQ is a brief 5-item instrument without subscales designed to measure perceived sleep quality in ICU settings using a visual analogue format (Richards et al., 2000). The Saudi validation (Al-Sulami et al., 2019) included 57 critically ill adult patients hospitalized in a university hospital in Jeddah (mean age 54 years; 61% male), whereas the Moroccan validation (Lkoul et al., 2025a) involved 224 ICU patients admitted to Hassan II University Hospital in Agadir (mean age 49 ± 18.3 years; 47.76% male).

The FSQ is a 27-item multidimensional instrument organized into four subscales designed to assess both sleep quality and environmental sleep disturbances in ICU patient (Freedman et al., 1999). The Moroccan validation included 260 critically ill adults (mean age 49.6 ± 18.4 years; 46.6% male) (Lkoul et al., 2025b) (Table 2).

Content validity was evaluated in all three ICU validation studies with “Very Good” methodological quality. The Moroccan studies (Lkoul et al., 2025a; Lkoul et al., 2025b) quantified expert agreement using the Aiken coefficient, showing high consensus for both the RCSQ (*V* = 0.82) and the FSQ (*V* = 0.78), complemented by pilot testing among ICU patients. The Saudi RCSQ adaptation (Al-Sulami et al., 2019) followed WHO translation guidelines and included cognitive debriefing interviews with 30 ICU patients to assess item clarity and comprehension. Based on these procedures, content validity was rated as sufficient (+) for all ICU instruments.

Structural validity was assessed in the Moroccan validation studies (Lkoul et al., 2025a; Lkoul et al., 2025b) using EFA with “Adequate” methodological quality. The Moroccan RCSQ (Lkoul et al., 2025a) showed a single-factor structure explaining 84.4% of the variance, while the Moroccan FSQ (Lkoul et al., 2025b) revealed a four-factor structure explaining 70.8% of the variance, consistent with its multidimensional design. Accordingly, structural validity was rated as sufficient (+) for these instruments. Structural validity was not evaluated in the Saudi RCSQ study (Al-Sulami et al., 2019), resulting in an inadequate rating for this property.

Internal consistency was assessed in all three studies with “Very Good” methodological quality. Cronbach’s alpha coefficients were 0.89 for the Saudi RCSQ (Al-Sulami et al., 2019), 0.96 for the Moroccan RCSQ (Lkoul et al., 2025a), and 0.82 for the Moroccan FSQ (Lkoul et al., 2025b), all exceeding the COSMIN threshold of 0.70. Therefore, internal consistency was rated as sufficient (+) for the ICU instruments.

Test–retest reliability was evaluated in the Moroccan validation studies (Lkoul et al., 2025a; Lkoul et al., 2025b) with “Very Good” methodological quality. The Moroccan RCSQ (Lkoul et al., 2025a) demonstrated excellent temporal stability (ICC = 0.978) over a six-hour interval, while the Moroccan FSQ (Lkoul et al., 2025b) showed ICC values ranging from 0.715 to 0.997 across its subscales. These results exceeded COSMIN thresholds, leading to a sufficient (+) rating. This property was not evaluated in the Saudi RCSQ study (Al-Sulami et al., 2019).

Construct validity was exclusively evaluated in the Moroccan RCSQ study (Lkoul et al., 2025a) with “Very Good” methodological quality. Convergent validity analyses demonstrated a significant inverse correlation between the RCSQ and PSQI total scores (*r* = −0.635), supporting the expected relationship between the instruments. Consequently, construct validity was rated as sufficient (+) (Tables 3–5).

(6) *Summary of overall findings*

Across the included studies, five Arabic-validated sleep quality instruments were identified: the PSQI, SQS, Single-item SQS, JSS, and ICU-specific instruments (RCSQ and FSQ). The PSQI was the most extensively validated instrument across diverse populations, whereas the other scales were evaluated in a limited number of studies. Internal consistency and structural validity were the most frequently assessed properties and were generally rated as sufficient. However, several measurement properties, including measurement error, responsiveness, and cross-cultural validity, were rarely evaluated across instruments.

## Discussion

This systematic review provides the first synthesis of translated and psychometrically validated Arabic sleep quality instruments. Using the PRISMA-COSMIN framework, 15 validation studies conducted across five Arab countries were identified. The findings reveal heterogeneity in both instruments and validation quality, with gaps in several psychometric properties.

(1) PSQI

Among the 15 included studies, the PSQI was the most frequently validated instrument in Arabic for assessing subjective sleep quality (*n* = 9), confirming its position as the most widely used sleep quality questionnaire (Mollayeva et al., 2016). Developed by Buysse et al. in 1989, the PSQI is a 19-item self-administered questionnaire designed to assess sleep quality over a one-month period in both clinical and non-clinical populations. It was conceptualized as a unidimensional measure comprising seven components contributing to a global score (Buysse et al., 1989). However, Arabic validations reveal a heterogeneous psychometric profile.

A central finding of this review is the discrepancy between methodological rigor and psychometric performance. Although most studies demonstrated strong methodological quality according to COSMIN criteria (Terwee et al., 2012), this did not consistently translate into sufficient measurement properties, indicating that appropriate validation procedures alone do not guarantee optimal psychometric performance across populations.

Internal consistency was evaluated in all nine studies, but only three achieved the COSMIN threshold (*α* ≥ 0.70) (Mokkink, n.d.), namely studies conducted among Jordanian patients with coronary artery disease (Suleiman et al., 2012), Omani cancer patients (Al Maqbali et al., 2020), and Saudi physicians (Malik et al., 2018). The remaining studies reported alpha values between 0.56 to 0.65, lower than those reported in the original validation (*α* = 0.83) (Buysse et al., 1989) and below the range typically reported in international adaptation (0.70–0.83) (Mollayeva et al., 2016). The meta-analysis (Mollayeva et al., 2016) suggested that lower alpha coefficients are frequently reported in non-clinical populations, particularly when certain components such as “use of sleep medication” exhibit floor effects in community samples.

However, the clinical versus non-clinical distinction does not fully explain the variability observed in Arabic validations. While acceptable internal consistency was found in two clinical populations (Suleiman et al., 2012; Al Maqbali et al., 2020), one satisfactory alpha value was also reported among Saudi physicians (Malik et al., 2018), a population not traditionally classified as clinical, but more frequently exposed to sleep disturbances and with greater access to pharmacological treatments (Liou et al., 2021; Francis et al., 2019). Greater variability in the “use of sleep medication” component may therefore increase inter-item correlations and overall reliability in this group.

Interpretation of reliability must also consider structural validity. Because Cronbach’s alpha assumes unidimensionality, reliability estimates depend on the factorial structure of the instrument (Mokkink, n.d.). Consistent with international findings summarized by Mollayeva et al. (2016), Arabic validation studies reported heterogeneous factorial structures, with some supporting a unidimensional model and others identifying two-factor solutions.

Importantly, adequate sample sizes were achieved in nearly all studies according to COSMIN recommendations (Mokkink, 2018), suggesting that factorial variability is unlikely to result from insufficient statistical power but may reflect population-specific perceptions of sleep quality.

Additional analyses further illustrate this variability. Rasch-based validation in healthy adults confirmed unidimensionality but reported low person reliability and disordered thresholds for several components (Aldhahi et al., 2026). Conversely, a two-factor solution in hemodialysis patients was associated with reduced internal consistency (Albatineh et al., 2022), whereas a similar multidimensional structure in cancer patients showed satisfactory reliability (Al Maqbali et al., 2020). These findings indicate that multidimensionality does not inherently reduce reliability, which also depends on factor cohesion and response distribution.

None of the Arabic PSQI validation studies evaluated criterion validity, a finding consistent with the meta-analysis by Mollayeva et al. (2016), which also reported no formal assessments of this property. This limitation largely reflects the absence of an established gold standard for measuring subjective sleep quality (Mollayeva et al., 2016). Although PSG, which simultaneously records brain activity, eye movements, muscle tone, respiration, and heart rate (Rundo and Downey, 2019), and actigraphy, based on accelerometer-derived movement recordings over extended periods to estimate sleep–wake patterns (Sujets ScienceDirect, 2008), are often considered reference techniques for sleep assessment, they primarily measures physiological sleep parameters and do not fully capture the subjective sleep experience targeted by the PSQI (Kline, 2013).

Overall, while the Arabic PSQI demonstrates generally adequate structural validity and acceptable reliability in selected populations, internal consistency results remain heterogeneous. Furthermore, measurement error, responsiveness, and cross-cultural measurement invariance were insufficiently examined. These findings suggest that although the PSQI remains the most established instrument in Arabic contexts, its psychometric profile is not uniformly optimal across populations.

(2) SQS and single item SQS

Beyond the PSQI, sleep quality assessment in general and occupational populations has also been addressed using two contrasting instruments: the comprehensive 28-item SQS and the ultra-brief Single-item SQS, illustrating the classic trade-off between measurement depth and practical feasibility.

The original SQS is a 28-item questionnaire designed to assess multiple dimensions of sleep quality (Yi et al., 2006). However, the Arabic validation (Alghamdi, 2025) identified a modified psychometric structure. Despite a rigorous translation process with expert review and pilot testing ensuring strong content validity, factor analyses did not support the original six-factor framework and instead revealed a five-factor model requiring the removal of one item. According to the authors, this structural change reflects culturally specific patterns in sleep perception, where dimensions such as “Satisfaction with Sleep” and “Restoration After Sleep” overlapped, while difficulties initiating and maintaining sleep were grouped into broader categories (Alghamdi, 2025). Similar structural variability has been reported internationally: while the original South Korean and Indonesian versions retained the six-factor structure (Semantic Scholar, n.d.), other adaptations, including the Chinese validation, produced reduced models (23 items, four factors) (Chen et al., 2021).

Consequently, while the Arabic SQS offers an acceptable psychometric structure, this reclassification raises questions regarding cross-cultural construct equivalence and highlights the importance of re-evaluating factorial structure during cross-cultural adaptation rather than imposing the original framework. Furthermore, while the Arabic instrument demonstrated strong global internal consistency (global Cronbach’s *α* = 0.88), variability across subscales (*α* ranging from 0.58 to 0.88) (Alghamdi, 2025) indicates that some dimensions may require further refinement.

These findings illustrate the limitations of lengthy multidimensional instruments in certain settings, which has led to the development of more pragmatic alternatives. Within this context, the Single-item SQS was introduced as a highly practical screening tool, asking respondents to rate their overall sleep quality on a single numerical scale (Snyder et al., 2018). Unlike the detailed 28-item SQS, this ultra-brief format was designed to minimize respondent burden in clinical and large-scale research settings (Snyder et al., 2018).

In its original development study, the Single-item SQS demonstrated strong psychometric performance, including high concurrent validity with the PSQI (*r* = −0.87 to −0.92) and significant correlations with the Morning Questionnaire Insomnia (MQI) (*r* = −0.76). It also discriminated effectively between normal sleepers and individuals with sleep disturbances and showed acceptable temporal stability (ICC = 0.74) while remaining sensitive to clinical change (Snyder et al., 2018).

The Arabic version was subsequently validated among Omani healthcare providers (Badahdah et al., 2024). From a COSMIN perspective, single-item instruments present methodological challenges, as structural validity and internal consistency cannot be evaluated. Consequently, psychometric evaluation relied on construct validity through hypothesis testing, which confirmed expected correlations with related constructs such as well-being (WHO-5) and anxiety (GAD-7) (Badahdah et al., 2024). These findings support the ability of the Single-item SQS to capture the core construct of subjective sleep quality while substantially reducing respondent burden. However, single-item measures inherently limit assessment of internal structure, measurement precision, and reliability, meaning they are suitable for rapid screening but cannot replace multidimensional instruments when detailed psychometric evaluation is required.

Taken together, these instruments offer complementary options depending on research objectives. The multidimensional 28-item SQS provides a detailed profile of sleep quality suitable for in-depth clinical or epidemiological research, whereas the Single-item SQS prioritizes feasibility, offering a rapid global indicator of sleep quality for large-scale screening or longitudinal monitoring.

(3) JSS

The JSS was originally developed as a brief instrument to assess the frequency of common nocturnal sleep complaints in epidemiological settings. Although it was not initially framed as a comprehensive “sleep quality” questionnaire, its total score is widely interpreted as an indicator of subjective sleep quality, with higher scores reflecting poorer perceived sleep (Jenkins et al., 1988).

The scale operationalizes sleep quality through four core symptoms (difficulty initiating sleep, nocturnal awakenings, early morning awakening, and non-restorative sleep) which are central components of how individuals subjectively evaluate their sleep experience (Jenkins et al., 1988). In this sense, the JSS conceptualizes poor sleep quality as the recurrent presence of insomnia-related complaints (Jenkins et al., 1988).

International validations have consistently supported its unidimensional structure and acceptable reliability, confirming the stability of this symptom-based operationalization across populations (Duruöz et al., 2018; Villarreal-Zegarra et al., 2022). The Arabic adaptation demonstrated strong methodological quality according to COSMIN standards and reproduced the expected structural and reliability properties, suggesting cross-cultural robustness of the construct measured (AlMashouk et al., 2024).

However, unlike comprehensive instruments such as the PSQI or the 28-item SQS, the JSS does not assess additional domains commonly associated with sleep quality, including sleep duration, efficiency, medication use, or daytime dysfunction (ScienceDirect Topics, 2020). Therefore, while the JSS provides a valid and efficient measure of perceived sleep quality based on nocturnal symptom frequency (Jenkins et al., 1988), it reflects a narrower conceptualization of the construct.

Compared with multidimensional instruments, the JSS offers greater structural stability but captures a narrower conceptualization of sleep quality, primarily centered on nocturnal symptom frequency.

For researchers and clinicians, the Arabic JSS may represent a pragmatic methodological compromise. Its brevity facilitates high response rates in large epidemiological studies and reduces respondent burden in clinical contexts, while still allowing for the evaluation of internal consistency and structural validity—psychometric properties that cannot be examined in single-item instruments.

Within the scope of this systematic review, the JSS appears to be a psychometrically promising tool for the rapid assessment of subjective sleep quality in general Arabic-speaking populations, particularly when sleep quality is conceptualized through the frequency of nocturnal disturbances.

(4) Instruments validated for ICU

Unlike conventional sleep quality instruments that assess sleep retrospectively over a defined recall period, sleep assessment in the intensive care unit must account for a fundamentally different and highly acute clinical context (Buysse et al., 1989). Sleep in the ICU is typically of poor subjective quality, highly fragmented, and often described as “atypical” (Dorsch et al., 2019). These unique characteristics necessitate instruments capable of evaluating sleep quality on a nightly basis within a highly disruptive environment. In this review, we identified two instruments specifically validated in Arabic for this context: the RCSQ, validated in Saudi Arabia (Al-Sulami et al., 2019) and Morocco (Lkoul et al., 2025a) and the -FSQ validated in Morocco (Lkoul et al., 2025b).

The RCSQ was originally developed with a pragmatic clinical objective, namely to provide a simple, rapid, and easily administrable measure of perceived sleep quality in critically ill patients (Richards et al., 2000). Its conceptual framework is based on a unidimensional representation of sleep quality, and the use of a five-item visual analogue scale, assessing sleep latency, sleep efficiency, sleep depth, number of awakenings, and overall sleep quality (Richards et al., 2000), reflects a deliberate methodological choice aimed at maximizing feasibility and clinical applicability in the demanding environment of ICU where patient burden is high and assessment time is limited (Watson et al., 2013).

During its initial development, the RCSQ demonstrated satisfactory psychometric properties, including good content validity, acceptable criterion validity against polysomnography, and high internal consistency (Cronbach’s *α* ≈ 0.90), supporting its construct validity within its intended clinical framework (Richards et al., 2000). These characteristics contributed to its widespread adoption in both clinical practice and research, and the instrument has since been translated and validated in several languages, including Arabic (Richards et al., 2020). In the Arabic validations, the RCSQ similarly showed excellent internal consistency and good clinical acceptability (Al-Sulami et al., 2019; Lkoul et al., 2025a; Lkoul et al., 2025b).

In contrast to the RCSQ, the FSQ is grounded in a broader and more analytical conceptual approach to sleep in ICU (Freedman et al., 1999). Its development was based on a multidimensional conceptualization of sleep, integrating not only subjective sleep perception but also the influence of environmental, organizational, and human-related factors inherent to the intensive care context (Freedman et al., 1999).

The FSQ was developed through a rigorous methodological process combining an extensive literature review, clinical expertise, and an analysis of sleep-disrupting factors specific to the intensive care environment (Freedman et al., 1999). This approach led to the construction of a multidimensional instrument comprising several items addressing complementary domains, including perceived sleep quality, sleep continuity, environmental disturbances (e.g., noise and light), care-related interruptions, and staff-related factors. Each item is rated on a numerical scale ranging from 1 to 10, allowing for a graded and clinically interpretable assessment of sleep and its determinants (Freedman et al., 1999).

In the Moroccan validation study (Lkoul et al., 2025b), the psychometric evaluation of the FSQ further supported the robustness of its conceptual framework. EFA identified a stable four-factor structure, reflecting the multidimensional nature of sleep disturbances in the intensive care setting. These factors accounted for a substantial proportion of the total variance, confirming the structural validity of the instrument. In addition, the Moroccan FSQ demonstrated good internal consistency with an overall Cronbach’s alpha coefficient = 0.816. Test–retest reliability was also found to be satisfactory (ICC: 0.715–0.997), indicating good temporal stability of the scores and supporting the instrument’s reliability for repeated measurements.

Taken together, these findings highlight two distinct but complementary approaches to the assessment of sleep quality in ICU. The RCSQ, designed as a brief and pragmatic screening tool, offers a rapid and easily applicable measure of perceived sleep quality (Richards et al., 2000), making it particularly suitable for routine clinical use and large-scale studies where feasibility is a primary concern (Richards et al., 2020). Its consistently high internal consistency and growing international use across different languages support its utility as a global indicator of sleep perception in critically ill patients (Richards et al., 2020). However, its unidimensional structure and reliance on subjective assessment limit its ability to capture the complexity of sleep disturbances in the ICU. In contrast, the FSQ grounded in a multidimensional approach and supported by more robust psychometric properties, appears better suited for research aimed at in-depth exploration of the mechanisms and determinants of sleep disturbances in critically. Ill patients (Freedman et al., 1999). The choice between these instruments should therefore be guided by the objectives of the study, the clinical context, and the level of methodological precision required.

Nevertheless, the absence of responsiveness and measurement error analyses limits conclusions regarding their sensitivity to clinical change in ICU settings.

### Cross-instrument comparison and conceptual considerations

Across all included instruments, a recurring pattern emerged: structural validity and internal consistency were the most frequently evaluated properties, whereas measurement error, responsiveness, and formal cross-cultural invariance analyses were rarely assessed. This imbalance reflects a broader trend in validation research, where reliability and factorial structure are prioritized over longitudinal performance and precision metrics.

Conceptually, the reviewed instruments illustrate varying operational definitions of sleep quality. Multidimensional scales such as the PSQI and SQS aim to integrate multiple domains of sleep experience, whereas shorter instruments such as the JSS and the Single-item SQS focus on core subjective perceptions. ICU-specific tools introduce context-dependent frameworks shaped by environmental and clinical factors. This heterogeneity underscores that “sleep quality” is not a uniform construct but rather a flexible concept influenced by theoretical orientation and clinical context.

Importantly, no single instrument demonstrated comprehensive evaluation across all COSMIN measurement domains. Therefore, instrument selection in Arabic-speaking populations should be guided not only by psychometric performance but also by conceptual alignment with the intended research or clinical objective.

### Strengths and limitations of the review

This review offers several strengths. First, it represents the first systematic synthesis specifically targeting Arabic-validated sleep quality instruments, addressing a critical gap in the literature. Furthermore, the application of the PRISMA-COSMIN framework ensured a rigorous, standardized appraisal of methodological quality and measurement properties, thereby minimizing subjective bias. Finally, the inclusion of instruments validated across diverse clinical, occupational, and critical care settings provides a comprehensive overview of how sleep quality is operationalized in the Arab world.

Several limitations must be acknowledged. First, the systematic search did not encompass grey literature, potentially introducing publication bias. Second, heterogeneity in study populations, psychometric methods, and instrument versions limited the comparability of findings and precluded the possibility of conducting a formal quantitative synthesis or meta-analysis of measurement properties. Finally, the conclusions drawn for certain instruments are constrained by the limited evaluation of key measurement properties, particularly responsiveness and measurement error, which restricts the ability to fully assess their longitudinal applicability. Despite these limitations, this review provides a structured and critical synthesis of the current state of Arabic-validated sleep quality instruments.

## Conclusion

Arabic sleep quality assessment has expanded considerably in recent years. The PSQI remains the most extensively studied instrument, although its psychometric performance demonstrates variability across populations. The SQS offers a multidimensional framework that requires structural adaptation in certain contexts, whereas the Single-item SQS and the JSS provide pragmatic alternatives that prioritize feasibility over structural complexity. Instruments developed for ICU, such as the RCSQ and the FSQ, address the unique clinical demands of critically ill populations.

Importantly, no single instrument demonstrated a comprehensive assessment across all COSMIN-recommended measurement domains. Future research should prioritize the evaluation of responsiveness, measurement error, and cross-cultural measurement invariance to strengthen the methodological robustness of these tools. A more systematic and comprehensive approach to validation will ultimately enhance the accuracy, comparability, and clinical utility of sleep quality measurement across diverse Arabic contexts.
